# Production and Isolation of Azaspiracid-1 and -2 from *Azadinium spinosum* Culture in Pilot Scale Photobioreactors

**DOI:** 10.3390/md10061360

**Published:** 2012-06-13

**Authors:** Thierry Jauffrais, Jane Kilcoyne, Véronique Séchet, Christine Herrenknecht, Philippe Truquet, Fabienne Hervé, Jean Baptiste Bérard, Cíara Nulty, Sarah Taylor, Urban Tillmann, Christopher O. Miles, Philipp Hess

**Affiliations:** 1 Ifremer, EMP/PHYC Laboratory, Rue de l'Ile d'Yeu, 44311 Nantes, France; Email: veronique.sechet@ifremer.fr (V.S.); philippe.truquet@ifremer.fr (P.T.); fabienne.herve@ifremer.fr (F.H.); sarah.taylor@myport.ac.uk (S.T.); 2 Marine Institute, Rinville, Oranmore, Co., Galway, Ireland; Email: jane.kilcoyne@marine.ie (J.K.); ciara.nulty@marine.ie (C.N.); 3 Nantes Atlantic University, MMS EA2160, 9 rue Bias, 44035 Nantes, France; Email: christine.herrenknecht@univ-nantes.fr; 4 Ifremer, BRM/PBA Laboratory, Rue de l'Ile d'Yeu, 44311 Nantes, France; Email: jean.baptiste.berard@ifremer.fr; 5 Alfred Wegener Institute, Am Handelshafen 12, D-27570 Bremerhaven, Germany; Email: urban.tillmann@awi.de; 6 Norwegian Veterinary Institute, P. O. Box 750 Sentrum, 0106 Oslo, Norway; Email: chris.miles@vetinst.no

**Keywords:** solid phase extraction, photobioreactor, chemostat, dinoflagellate, micro-algae, LC-MS/MS, tangential flow filtration, azaspiracid, HP-20

## Abstract

Azaspiracid (AZA) poisoning has been reported following consumption of contaminated shellfish, and is of human health concern. Hence, it is important to have sustainable amounts of the causative toxins available for toxicological studies and for instrument calibration in monitoring programs, without having to rely on natural toxin events. Continuous pilot scale culturing was carried out to evaluate the feasibility of AZA production using *Azadinium spinosum* cultures. Algae were harvested using tangential flow filtration or continuous centrifugation. AZAs were extracted using solid phase extraction (SPE) procedures, and subsequently purified. When coupling two stirred photobioreactors in series, cell concentrations reached 190,000 and 210,000 cell·mL^−1^ at steady state in bioreactors 1 and 2, respectively. The AZA cell quota decreased as the dilution rate increased from 0.15 to 0.3 day^−1^, with optimum toxin production at 0.25 day^−1^. After optimization, SPE procedures allowed for the recovery of 79 ± 9% of AZAs. The preparative isolation procedure previously developed for shellfish was optimized for algal extracts, such that only four steps were necessary to obtain purified AZA1 and -2. A purification efficiency of more than 70% was achieved, and isolation from 1200 L of culture yielded 9.3 mg of AZA1 and 2.2 mg of AZA2 of >95% purity. This work demonstrated the feasibility of sustainably producing AZA1 and -2 from *A. spinosum* cultures.

## 1. Introduction

**Figure 1:**
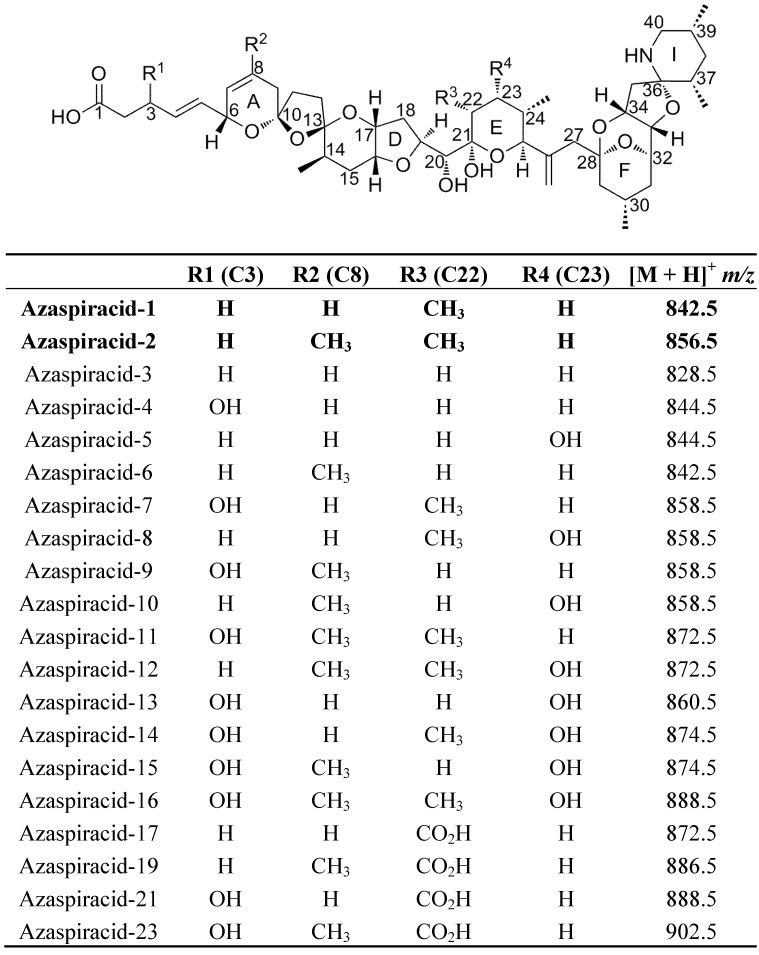
Azaspiracid (AZA) structures and mass-to-charge ratios (*m*/*z*) for the molecularions [M + H]^+^ detected in *A. spinosum* and in mussels (*M. edulis*). Toxins found in *A. spinosum* are shown in bold text.

Four groups of lipophilic marine algal toxins are currently regulated in Europe; among which azaspiracids (AZA) is the most recently discovered group of toxins. In 1995, the first human intoxication with AZA occurred in the Netherlands after consumption of contaminated mussels from Ireland, with symptoms similar to diarrhetic shellfish poisoning (nausea, stomach cramps, vomiting and diarrhea) [1]. A few years later, the toxin was named AZA following identification and isolation from contaminated shellfish [2], and the structure was subsequently revised thanks to synthetic studies [3]. Afterwards, a number of analogues were discovered in mussel tissues using biological assays and chemical analysis, including mass spectrometric techniques, *i.e.*, AZA2-32 [4,5,6,7,8,9]. Nevertheless, since the first known poisoning event, twelve years passed until the discovery of a primary producer, the dinoflagellate *Azadinium spinosum* (strain 3D9) [10,11,12]. This small dinoflagellate (12–16 µm length and 7–11 µm width) produces AZA1 and -2 in culture (Figure 1) [12]. Since this recent discovery, the new genus *Azadinium* has been encountered in different parts of the world (Ireland [13], Mexico [14], Argentina [15] and Korea [16]). Furthermore, azaspiracids were found in Europe, America, North Africa, and Asia [17,18,19,20,21,22,23], and AZA events are now recognized as a world-wide phenomenon [23]. Interestingly, the AZAs known to be implied in food poisoning have not been shown to be produced by species other than *A. spinosum* (and by metabolism of AZA1 and -2 in *Mytilus edulis*), e.g., *Azadinium obesum* [24] and *Azadinium poporum* [16,25]. However, Krock *et al*. [26] recently foundcompounds structurally related to AZAs in a culture of *A. poporum*, and confirmation of the toxicity of these compounds will be important to assess the homogeneity of the genus with regards to toxin production and structure-activity.

Currently, the main sources of marine algal toxins for purification are derived from producing organisms in culture or harvested from natural blooms (e.g., okadaic acid group toxins [27], brevetoxins [28], saxitoxins [29], yessotoxins [30], cyclic imines [31,32,33] and pectenotoxins [34]), from contaminated shellfish [35,36], or from bulk extraction of environmentally contaminated HP-20 resin [37]. Isolation from the producing organism is preferred, as extracts are considerably purer than shellfish extracts and their availability is not dependent on the occurrence of natural toxic episodes.

Purification of phycotoxins is essential as there is currently a shortage of pure calibration and reference materials for phycotoxin monitoring in food [38]. This has become particularly important as micro-algal lipophilic toxins in contaminated shellfish are now monitored in Europe using LC-MS/MS as a reference method [39], and AZA analysis by LC-MS/MS requires purified AZA standards for quantitation. Naturally occurring *A. spinosum* blooms are hard to predict and/or find, as very little data are available on its life history. The organism is small and hard to differentiate under light microscopy from other small dinoflagellates such as *Heterocapsa* and similar species. These difficulties hinder identification of such blooms and prediction of subsequent shellfish contaminations that could be used for the necessary purifications. Sustainable production of toxins from *A. spinosum* culture would thus be desirable for instrument calibration in monitoring programs and for toxicological studies.

**Figure 2:**
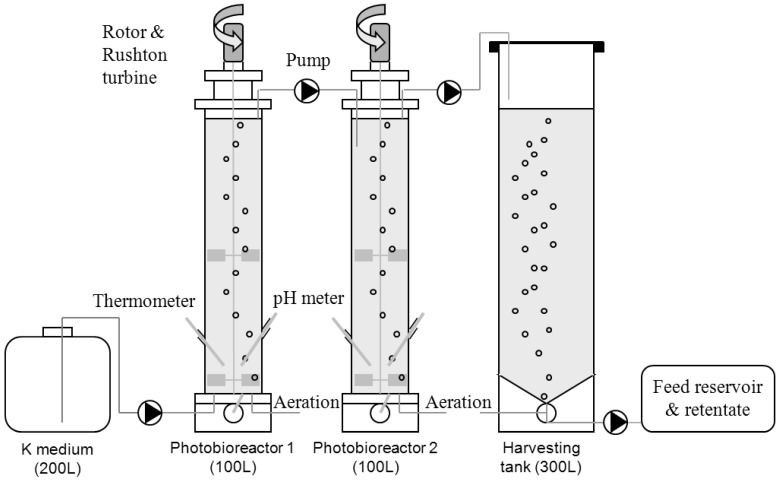
Schematic representation of *A. spinosum* and AZA production system using photobioreactors in series.

The aim of this study was to evaluate the feasibility of azaspiracid production from *A. spinosum* in pilot scale photobioreactors. In previous studies, a continuous system was developed [40,41]. Two stirred photobioreactors were coupled in series (Figure 2) to assess how dilution rate influences cell concentration as well as toxin production. To harvest toxin, we applied a dual approach for the recovery of AZAs from both cultured cells and from the culture supernatant. For the recovery of cells, tangential flow filtration and continuous centrifugation were evaluated. Solid phase extraction procedures were developed to recover AZAs from large volumes of *A. spinosum* culture supernatant and from concentrated cell suspensions. A method developed to purify AZA1 and -2 from crude algal extract is presented, highlighting the effectiveness of this purification procedure compared to purification of AZA1 and -2 from mussel digestive glands.

## 2. Results and Discussion

*A. spinosum* (strain 3D9) was received at Ifremer through collaboration with the Alfred Wegener Institute, as part of the ASTOX2 project. After receipt of the organism, preliminary studies on environmental and nutritional factors were conducted to allow the organism to grow in large-scale culture, and to increase cell concentration and toxin production (data not shown). Even though dinoflagellates are usually considered sensitive to shear stress produced by small-scale turbulence [42], it was possible to grow this species in stirred 100 L photobioreactors in series.

### 2.1. Effect of Dilution Rate on *A. spinosum* and AZA Production in Pilot Scale Photobioreactors

Because cultures collapsed when the dilution rate was altered significantly within one experimental run, separate independent experiments were conducted at different dilution rates. At a dilution rate of 0.1 day^−1^, steady state cell concentration was lowest of all dilution rates studied (174,000 and 164,000 cell·mL^−1^ in reactor 1 and -2 (R1 and R2), respectively) (Table 1). Cell concentration, mean estimated spherical diameter (ESD), cellular volume and toxin content were stable at steady states for dilution rates from 0.15 to 0.30 day^−1^. For all dilution rates from 0.15 to 0.3 day^−1^, steady state cell density only increased by about 10% between R1 and R2 (190,000 and 215,000 cell·mL^−1^, respectively). Results for ESD and cellular volume were comparable at all dilution rates but differed significantly between R1 and R2. Even though, the cell density increased only by about 10% between R1 and R2, the cellular volume increased by about 50%. The small increase in cell density (*or* concentration) and the large increase in bio-volume reflect that cell division mainly takes place in the first reactor while increase in cellular volume with little cell division occurs in the second reactor. While the physiological limitation leading to this phenomenon in each reactor is not yet fully understood, the increase in cell-volume is referred to as cell maturation from here on.

At each steady state studied, the AZA cell quota (*i.e.*, AZA1 + 2) increased between R1 and R2, reflecting that during maturation of cells in the second bioreactor, cells continued to produce AZAs. The increase in toxin per cell from R1 to R2 reached its maximum at a dilution rate of 0.3 day^−1^ (ratio of 2.6 between cell quota in R2 compared to R1). The toxin production of cells severely limited in growth suggests that AZAs can be considered to be secondary metabolites not implicated in cell division or cell growth processes, as has been suggested for polyketides and many toxins in general [43,44]. Consequently, in contrast to cell density, AZA per cell decreased as dilution rate increased from 0.15 to 0.3 day^−1^, ranging from 67 to 24 fg·cell^−1^ for R1 and 98 to 63 fg·cell^−1^ for R2. This decrease in AZA per cell was partly counteracted by the maturation effect and by the fact that at higher growth rates more cells are produced per day. Thus, as cell numbers increased with dilution rate, and as the cell volume still always increased in the second bioreactor, AZA production reached an optimum of 475 ± 17 µg·day^−1^ at a flow rate of 25 L·day^−1^.

In the harvesting tank, at a dilution rate of 0.1 day^−1^, cell density collapsed before harvesting at a concentration of 70,000 cell·mL^−1^. However, at the other dilution rates, cell concentration and AZA cell quota were maintained in the harvesting tank until 200 L culture volume was reached. At a dilution rate 0.2 day^−1^, a decrease of 10% in cell concentration and AZA cell quota was observed in the harvesting tank compared to R2. At dilution rate 0.25 day^−1^, a slightly smaller decrease of 0–5% in cell concentration and AZA cell quota was observed while an increase of 0–5% was observed at 0.3 day^−1^ (0.15 day^−1^ was not evaluated but culture did not collapse). 

Previous studies on batch cultures of *A. spinosum* (strain 3D9 or SM2) showed a production of AZA1 and -2, with AZA1 as the predominant AZA and with a cell quota ranging from 5 to 50 fg·cell^−1^ [12,13]. In the present study, the same toxin profile was found, however, AZA cell quotas of 24–98 fg·cell^−1^ were obtained depending on dilution rate. These results raised the possibility of improving both cell concentration and toxin concentration in *A. spinosum* grown in bioreactors by controlling dilution rate (equivalent to growth rate in chemostat bioreactors) and by adding a period of maturation using a second bioreactor specifically dedicated to improving the AZA cell quota.

**Table 1:** *A. spinosum* concentration (cell·mL^−^^1^), mean estimated spherical diameter (ESD) (µm), cellular volume (µm^3^·mL^−^^1^), toxin content (fg·cell^−^^1^), and cell and toxin production (cell·day^−^^1^ and µg·day^−^^1^, respectively) at the dilution rates studied (0.1, 0.15, 0.2, 0.25, 0.3 day^−^^1^) in the two bioreactors in series (R1 and R2). Standard deviations were calculated from sequential repeat measurements of each culture and the last columns present the result of the multifactorial ANOVA followed by a Fisher least-significant-difference test to discriminate differences between values within each factor.

*A. spinosum*	*n*	0.1 day^−1^		0.15 day^−1^		0.2 day^−1^		0.25 day^−1^		0.3 day^−1^		Main factors		Interaction
		R1	R2	R1	R2	R1	R2	R1	R2	R1	R2	Dilution rate (D)	Reactor (R)	D–R
Concentration(×10^3^ cell·mL^−1^)	9–22	174 ± 6	164 ± 4	193 ± 6	214 ± 3	194 ± 8	214 ± 7	190 ± 6	221 ± 5	187 ± 5	220 ± 4	*p* ≤ 0.05	*p* ≤ 0.05	*p* ≤ 0.05
												0.1 < 0.15 = 0.2 = 0.25 = 0.3	R1 < R2	
Mean ESD(µm)	9–22	9.81 ± 0.09	10.1 ± 0.1	9.59 ± 0.15	9.9 ± 0.2	9.6 ± 0.2	10.1 ± 0.2	9.29 ± 0.09	9.93 ± 0.04	9.5 ± 0.1	10.02 ± 0.05	*p* ≤ 0.05	*p* ≤ 0.05	*p* ≤ 0.05
												0.25 = 0.3 < 0.15 < 0.2 = 0.1	R1 < R2	
Cellular volume(×10^7 ^µm^3^·mL^−1^)	9–22	8.8 ± 0.4	8.4 ± 0.3	9.2 ± 0.4	11.1 ± 0.4	9.3 ± 0.6	12.0 ± 0.7	8.2 ± 0.3	11.7 ± 0.2	8.5 ± 0.1	12.0 ± 0.4	*p* ≤ 0.05	*p* ≤ 0.05	*p* ≤ 0.05
												0.1 < 0.25 = 0.3 = 0.15 < 0.2	R1 < R2	
AZA1 (fg·cell^−1^)	3–5 *	37 ± 5	65 ± 8	52 ± 6	74 ± 4	34 ± 12	76 ± 14	26 ± 2	61 ± 3	17 ± 1	45 ± 3	*p* ≤ 0.05	*p* ≤ 0.05	*p* ≤ 0.05
AZA2 (fg·cell^−1^)	3–5 *	10 ± 1	16 ± 1	15 ± 1	24 ± 2	10 ± 2	19 ± 2	12 ± 2	25 ± 2	7 ± 1	18 ± 2			
AZA1 + 2 (fg·cell^−1^)	3–5 *	47 ± 6	81 ± 9	67 ± 3	98 ± 5	44 ± 13	95 ± 16	38 ± 2	86 ± 3	24 ± 1	63 ± 5	0.3 < 0.25 = 0.1< 0.2 < 0.15	R1 < R2	
Cell production (×10^9^ cell·day^−1^)	n/a	1.74 ± 0.06	1.64 ± 0.04	2.90 ± 0.09	3.21 ± 0.05	3.9 ± 0.2	4.3 ± 0.1	4.8 ± 0.2	5.5 ± 0.1	5.6 ± 0.2	6.6 ± 0.1	Cell production = Cell concentration × D		
Toxin production AZA1 + 2 (µg·day^−1^)	n/a	82 ± 3	134 ± 15	193 ± 9	314 ± 15	170 ± 50	406 ± 64	180 ± 10	475 ± 17	134 ± 5	415 ± 33	Toxin production = Cell production × [AZA1 + 2]		

* Each sample was also injected in triplicate to reduce analytical variability; n/a not applicable.

Preliminary studies on *A. spinosum* in batch culture gave cell concentrations up to ~90,000 cell·mL^−1^ (data not shown) and toxin quotas of 5 to ~50 fg·cell^−1^ [12,13]. Using a photobioreactor, both cell concentration and AZA cell quota were each increased by a factor of 2 compared to batch culture without aeration. Even though batch culture with aeration gave a similar cell concentration (data not shown), our study demonstrated the advantage of continuous culture for *A. spinosum*, due to the control of both cell concentration and toxin production, minimizing the risk of culture collapse. Hence the process developed ensures a continuous production of *A. spinosum* for AZAs isolation and purification.

Reported AZA cell quota (previous work and our own data) for *A. spinosum* in the femtogram-range are low compared to toxin quota of other dinoflagellates, which are in the picogram-per-cell range, e.g., *Alexandrium* [45], *Dinophysis* [46], *Prorocentrum* [47], *Karenia* [48] and this seemed to be discouraging for toxin production. However, considering the small size of *A. spinosum* cells, toxin concentrations calculated on a per volume basis (0.23 fg·µm^3^) were similar to those of other toxic dinoflagellates such as *Dinophysis acuta* (0.89–1.1 fg·µm^3^, calculated from Pizarro *et al.* [49]).

Even though published results for toxin production by other dinoflagellates are encouraging (*Alexandrium minutum* ~20 µg toxin·L^−1^·day^−1^ [50], similar to *A. spinosum*; *Protoceratium reticulatum* 214 µg·L^−1^·day^−1^ [51], 10 times more than to *A. spinosum*; *Alexandrium ostenfeldii* (higher cell concentration but lower toxin cell quota—71,000 cell·mL^−1^, 0.7 pg·cell^−1^ 13-desMeC SPX eq, than in batch culture ~17,000 cell·mL^−1^, 4.2 to 1.7 pg·cell^−1^ 13-desMeC SPX eq [45] (SPX = Spirolide). More effort should be made to optimize toxin production, since, most toxins produced by harmful dinoflagellates are required for instrument calibration, and further research is required on toxicology and other biomedical aspects. Furthermore, studies on dinoflagellates in bioreactors would help in understanding their complex physiology and the link between growth and toxin production.

The work presented here indicates that *A. spinosum* can grow in stirred photobioreactors, even though it is a small and fragile dinoflagellate. The study shows the effect of growth rate on AZA cell quota, thus contributing to the understanding of the biosynthetic behaviour of *A. spinosum*. Furthermore, the set-up developed may be used to assess more generally ecophysiological characteristics of dinoflagellates.

### 2.2. Separation Procedure of *A. spinosum* from the Culture Medium

AZA extraction procedures were developed to optimize AZA recovery from the *A. spinosum* pilot-scale culture. Two methods were tested, tangential flow filtration and continuous centrifugation.

In the harvesting tank, *i.e.*, prior to large-scale separation of cells from culture medium, 95 ± 4% of the AZA1 and -2 present were intra-cellular, as estimated by toxin quantification in both algal pellet and supernatant. However, after tangential flow filtration (method 1), 50 to 70% of the toxin was in the concentrate and 30 to 50% had been released into the permeate. Toxin release was time-dependent, with longer filtration times leading to higher proportions of toxin in the permeate (depending on age of culture and cartridge). This procedure allowed for the separation of 200 L of culture into 0.7–1.0 L of algal concentrate (equivalent to 40–46 g of wet algal paste after centrifugation) and ~200 L of permeate. However, the release of a non-negligible amount of dissolved AZAs into the permeate necessitated the development of AZA adsorption procedures to avoid significant AZA losses. Interestingly, after tangential flow filtration, the algal cells retained their integrity and the permeate was clear, demonstrating the weak effect of shear forces on *A. spinosum* cells.

In contrast to tangential flow filtration, continuous centrifugation (method 2) led to significant lysis of algal cells (probably due to the violent release of concentrate from the centrifuge), and few entire cells were observed microscopically. This last procedure allowed for the separation of 200 L of culture medium into ~15 L of algal concentrate and ~185 L of permeate containing, respectively, 76% ± 7% and 24% ± 1% of the toxin in each fraction. Continuous centrifugation was less time consuming than tangential flow filtration and can easily be scaled up to much larger volumes of culture. However, the initial purchase cost of centrifugal equipment is higher than for tangential flow filtration, and it was not possible to recover an algal paste under the test conditions using continuous centrifugation. The choice of cell recovery technique therefore depends on financial resources and other research needs.

### 2.3. AZA1 and -2 Extraction from the Retentate and Permeate

After tangential flow filtration, the majority of toxin from the concentrated cells was recovered in the algal paste after centrifugation of the retentate (method 3), with only minor loss of AZAs into the retentate supernatant (9 ± 1%).

Three organic solvents were tested to assess extraction yield and purity (method 3): acetone, acetonitrile (ACN), and dichloromethane (DCM). No differences were observed between the three solvents (Table 2), confirming results previously obtained during *A. spinosum* analysis [52]. Methanol was not used as it was shown to induce AZA extraction artifacts when used as solvent for *A. spinosum* analysis [52]. Significant differences in purity were observed (Table 2), with acetone producing more residue than ACN and DCM. These procedures were also compared with adsorption on Diaion HP-20 polymeric resin (adaptation of method 4). The resin gave comparable extraction efficiency to solvent extraction, but with significantly better selectivity (*i.e.*, purity) (Table 2).

**Table 2:** Azaspiracid yield (µg·g^−1^ ± SD, *n* = 3) and purity (%) from algal paste after extraction with acetone, ACN or DCM (method 3), and using HP-20 resin.

	Acetone	ACN	DCM	HP-20
AZA1 + 2 (µg·g^−1^)	17.4 ± 0.5	18 ± 2	17 ± 1	17 ± 1
Purity (%)	0.036 ± 0.002	0.07 ± 0.01	0.09 ± 0.01	0.21 ± 0.03

To recover AZAs from the permeate and retentate-supernatant, representing up to 50% of the total AZAs, solid phase adsorption procedures were implemented using HP-20 resin (methods 5 and 6). The HP-20 polymeric resin was previously used and studied by MacKenzie *et al.* [53] and Fux *et al*. [54,55,56] for adsorption of lipophilic toxins. The study by Fux *et al*. and Rundberget *et al*. demonstrated that HP-20 resin is a suitable adsorbent for AZAs [37,56]. In the present study, HP-20 resin was used for adsorption of AZA, with volumes ranging from 0.75 L of algal concentrate to 200 L of permeate. Method 4 was developed to recover both the intra- and extra-cellular toxins from the retentate. The results of this work are summarized in Table 3.

To optimize extraction yield, four separate experiments (using different lots of concentrate) were carried out to determine: (1) the volume of solvent; (2) the optimum amount of resin to be used; (3) the time of contact between the sample and the resin; and (4) the method for AZA desorption from the HP-20 resin.

**Table 3:** Azaspiracid yield (µg/mL of concentrate or %, ± SD, *n* = 3) using various HP-20 adsorption and elution procedures (method 4) *.

Acetone Volume/5 g HP-20	3 × 5 mL		3 × 10 mL		3 × 25 mL		
AZA yield (µg/mL)	1.61 ± 0.07		2.1 ± 0.1		2.19 ± 0.06		
**Mass HP-20/100 mL concentrate**	**1 g**		**2.5 g**		**5 g**		
AZA yield (µg/mL)	2.6 ± 0.2		2.6 ± 0.1		2.1 ± 0.2		
**Time of contact**	**2 h**	**6 h**		**24 h**		**72 h**	
AZA yield (µg/mL)	0.48 ± 0.06	0.52 ± 0.03		0.61 ± 0.02		0.52 ± 0.08	
HP-20 adsorption efficiency (% after 24 h of contact with the concentrate)	93.8 ± 0.1						
**Desorption procedure (2.5 g HP-20–3 × 7.5 mL acetone)**	**Soaking using acetone and filtration**				**Column using acetone **		
Time of soaking (a and b), and flow rate (c)	**(a) 5 min**		**(b) 2 h**		**(c) 1 mL·min** ^−**1**^		
Fraction 1 (%)	77 ± 3		74 ± 5		98.2 ± 0.5		
Fraction 2 (%)	21 ± 1		22 ± 3		1.6 ± 0.8		
Fraction 3 (%)	3.0 ± 0.4		3.8 ± 0.2		0.2 ± 0.1		
AZA yield (µg/mL)	2.43 ± 0.09		2.4 ± 0.1		2.58 ± 0.01		
**Desorption yield (%)**	**83 ± 3**		**81 ± 4**		**88.5 ± 0.2**		
**Total extraction yield (%)**	**78 ± 3**		**76 ± 4**		**83.1 ± 0.1**		

* A separate lot of concentrate was used to determine each parameter, so yields are only comparable within each experiment.

Smaller resin masses (1 and 2.5 g) gave better extraction yield after 24 h of contact (Table 3). 2.5 g of HP-20 resin for 100 mL of retentate was chosen to avoid difficulty of elution and to ensure sufficient adsorption capacity of the resin in case of high AZA concentration.

Results showed that, to reach the optimum desorption yield a minimum of six times the HP-20 resin volume of extracting solvent was required. The best results of AZA desorption from the resin were obtained using a glass column with three successive elutions with acetone at 1 mL·min^−1^. This last procedure eluted 98.2% ± 0.5% of the toxin recovered in the first elution volume, showing a better desorption than with the other procedures. Using the optimized procedure, a final yield of 83.1% ± 0.1% of the initial amount of AZA1 + 2 in the algal concentrate was obtained. The yield was close to that obtained by Fux *et al.* for AZA1–3 (85%–93%) [55], but is lower than for other lipophilic toxins [53,55].

Extracellular toxin was extracted using two separate procedures, each with HP-20 (methods 5 and 6). SPATT bags were initially designed as a monitoring tool to follow and predict micro-algal toxic events around shellfish production areas [53,57]. The procedure using HP-20 packed in a column as a solid phase extraction method was implemented for biotoxin extraction from seawater after naturally occurring microalgal blooms [37]. These two methods were adapted to laboratory conditions after tangential flow filtration of the culture medium to recover dissolved AZA from the permeate (method 5 and 6).

Both methods gave similar recovery (~80%–85% of dissolved toxins); however, recovery using SPATT bags (method 5) showed more variability than the SPE procedure (method 6, Table 4). 

**Table 4:** AZA mass balance (% ± SD, *n* = 3 for methods 4 and 6, and *n* = 7 for methods 3 and 5) obtained after tangential flow filtration using extraction methods 3–6 (indicated in parentheses). Recovery was calculated from the sum of AZA1 + 2 concentration measured in the harvesting tank before tangential flow filtration.

Method No.	Method description	% Recovery of total
(3)	Algal paste	56 ± 9
(4)	Algal retentate + HP-20	54 ± 3
(5)	Algal permeate + SPATT	21 ± 9
(6)	Algal permeate + SPE	26 ± 4

Finally, these optimized procedures allowed us to recover ~3 mg of AZAs in crude extracts after tangential flow filtration of 200 L of culture. This crude extract was obtained in 12 working days, with 8 days of culturing (at steady state) at a flow rate of 25 L·day^−1^, 1 day (between 5 and 8 h work) of filtration, and 2–3 days of extraction.

### 2.4. Isolation of AZA1 and -2 from *A. spinosum* Crude Extract

Isolation of AZAs from contaminated shellfish was previously performed using a 7-step procedure [36]. However, *A. spinosum* extracts obtained using HP-20 were considerably purer (0.5% w/w, on average, for pilot-scale recovery, line 1 Table 5) than the shellfish extracts used in the study of Kilcoyne *et al*. [36]. Hence, it was expected that fewer steps would be required to purify AZA1 and -2 to obtain certified reference standards and to perform toxicological studies. As with the isolation from shellfish, the crude extract was initially partitioned between ethyl acetate and 1 M sodium chloride. The step resulted in a clean-up of 57% and a recovery of 90%. Previous studies have shown that AZAs are unstable in acidic environments, and that increased temperature will accelerate any acid-catalysed degradation of AZAs [58]. However, small scale purification trials by chromatography on a silica gel column showed that it was safe to have acetic acid in the eluent at this point of the procedure. Thus, as also demonstrated by Perez *et al.* [35] and Kilcoyne *et al.* [36], the sample is still quite crude at this stage of the isolation, and hence the remaining matrix exerts a similar protective effect to that of shellfish tissue [58]. Silica gel chromatography (step 2) gave a very high efficiency in terms of clean up (87%) and recovery (~91%, Table 5).

**Table 5:** Batch summary table for purification of AZA1 and AZA2.

Step No.	Step	AZA1 (mg)	AZA2 (mg)	Weight (g)	Purity (%) ^†^
	HP-20 resin extract	12.5	3.2	3.04	0.5
1	Partitioning	11.2	3.0	1.32	1.1
2	Silica gel	10.2	2.8	0.17	7.6
3	Flash (Phenyl-Hexyl) *	9.7	2.4	0.01	>90
4	Prep HPLC (C8/C18)	9.3	2.2	-	>95
	% Recovery (steps 1–4)	75	70		

* AZA1 and AZA2 were separated from each other in this step. ^†^ Total AZA1 + 2, based on w/w.

The third step in the procedure employed flash chromatography using a phenyl-hexyl stationary phase and a weakly alkaline mobile phase to prevent degradation of toxins. Separation of AZA1 and -2 was achieved in this step with separate fractions being collected. This step resulted in the highest clean-up (93%) and recovery (95%) (Table 5).

Final purification was achieved by semi preparative HPLC using a neutral mobile phase. Fractions were collected based on UV detection (210 nm) to prevent contamination from other non-AZA analytes. This step resulted in recoveries of ~93% while clean-up is minimal at this stage.

SPE cartridges were used to remove any buffer remaining in the sample, as well as to reduce the water content in, and volume of, the AZA fractions prior to evaporation, and as an additional final clean-up step to remove trace contaminants introduced via the LC eluents. This SPE procedure resulted in very little loss of toxin, with recoveries of >95% being achieved, and greatly facilitated evaporation of the purified AZA-fractions to dryness. The ^1^H NMR spectra of AZA1 and -2 were compared to published NMR data and found to be identical. Examination of the spectra indicated purities of >95%.

Purified AZA1 (9.3 mg) was obtained along with 2.2 mg of AZA2. Overall recoveries (steps 1–4) were 75% for AZA1 and 70% for AZA2; ^1^H NMR spectra of AZA1 and -2 following purification from *A. spinosum* are presented in electronic supplementary information (ESM) to demonstrate purity. This recovery is a significant improvement compared to isolations from shellfish with recoveries increasing by a factor of ~1.5. Furthermore, the procedure is significantly easier to perform with two fewer clean-up steps after extraction being required to achieve sufficient purities.

## 3. Experimental Section

### 3.1. Culture Conditions and Measurement

Strain 3D9 of *A. spinosum* was grown in two stirred photobioreactors of 100 L (1827 mm × 300 mm) operated in series and tested in separate experiments at the following dilution rates (0.1, 0.15, 0.2, 0.25 and 0.3 day^−1^ per bioreactor). Culture was collected in an aerated harvesting tank (300 L), maintained at 18 °C (Figure 2). The culture medium was a K-modified medium [59], without NH_4_Cl and tris base but with Na_2_SeO_3_ enrichment (10^−8^ M).

Prior to inoculation, photobioreactors were sterilized using peroxyacetic acid (5 ppm) for 30 min. The first photobioreactor was inoculated with 30 L of *A. spinosum* culture at 70,000 cell·mL^−1^ and filled up at the indicated dilution rate to 100 L before filling the second photobioreactor by pumped transfer at the same flow rate.

The photobioreactors were made of transparent polymethyl methacrylate and operated using the following conditions: the pH was maintained at 7.9 using CO_2_ addition and the temperature at 18 °C by adjusting room temperature to maintain 18 °C inside the bioreactors (automatic feed-back). Light was provided on one side of the reactor using neon tubes with a photon flux density at 200 µmol·m^−2^·s^−1^, and a photoperiod of 16 h of light and 8 h of dark. A Rushton turbine was used to stir the culture at 40 rpm.

Cell concentration (cell·mL^−1^), average cell size (estimated spherical diameter, µm) and total cellular volume (µm^3^·mL^−1^) were assessed daily using a particle counter (Multisizer 3 Coulter counter, Beckman). The bioreactors were considered to have reached steady state after a minimum of five days at the same micro-algal concentration (±5%).

### 3.2. *A. spinosum* Analysis

When steady state was achieved, triplicate samples of *A. spinosum* were taken from each bioreactor to assess toxin content daily over one week; the same analyses were carried out from the 300 L harvesting tank before each tangential flow filtration for initial toxin content assessment.

The analytical procedure has previously been optimized [52]. Briefly, aliquots (10 mL) of *A. spinosum* culture were collected and centrifuged (2500 *g*, 20 min, 4 °C) in 15 mL tubes. The supernatant was collected (for extra-cellular toxin content) and the pellet was re-suspended in 0.5 mL of acetone–water (9:1, v/v), transferred to an Eppendorf tube (1.5 mL) and bath-sonicated (10 min). After sonication, the aliquot was centrifuged (15,000 *g*, 10 min, 4 °C). The supernatant was transferred to a 5 mL glass tube and gently evaporated under nitrogen on a heating block at 35 °C. This process was repeated so that the pellet was extracted three times in total. After evaporation of supernatants, the residue was reconstituted in 1 mL methanol. An aliquot was filtered with Nanosep MF centrifugal filter 0.2 µm (Pall) (15,000 *g*, 3 min, 4 °C), and transferred to an HPLC vial for analysis.

The aqueous supernatant from centrifugation of the algal culture was transferred to a 15 mL glass tube and 5 mL of DCM was added. The mixture was homogenized and centrifuged (2500 *g*, 10 min, 4 °C). The organic phase was transferred to a 15 mL glass tube and gently evaporated under nitrogen on a heating block at 35 °C. The aqueous phase was extracted three times in this manner, and following evaporation, the residue was reconstituted and filtered as above.

This last procedure was also used to estimate AZA concentration in the algal retentate and to assess AZA adsorption by the HP-20 resin.

### 3.3. Solid Phase Extraction Procedure

SPE was carried out on Oasis HLB cartridges (Waters) to estimate AZA concentration in 50 mL samples of the permeate and to evaluate AZA adsorption kinetics by the HP-20 resin. Oasis HLB cartridges, (6 cc, 200 mg) were activated with methanol (10 mL) and washed with a solution of water–methanol (9:1 v/v, 10 mL). The sample was loaded dropwise. Once loaded, the cartridge was washed with a solution of water–methanol (9:1 v/v, 10 mL). The sample was eluted with 5 mL of methanol into a glass tube and gently evaporated under nitrogen on a heating block at 35 °C to yield a residue which was reconstituted in 0.5 mL methanol and filtered as above [36].

### 3.4. Separation of *A. spinosum* from the Culture Medium

*Method 1*. Tangential flow filtration to separate the algae from the culture medium was based on Sartorius crossflow filtration using five Hydrosart 0.1 m^2^ open-channel microfiltration cassettes (Sartorius Stedim Biotech) mounted in a stainless steel holder (Sartorius Stedim Biotech) with a 4-piston diaphragm pump (Jabsco, SartoJet-Pump).

**Figure 3:**
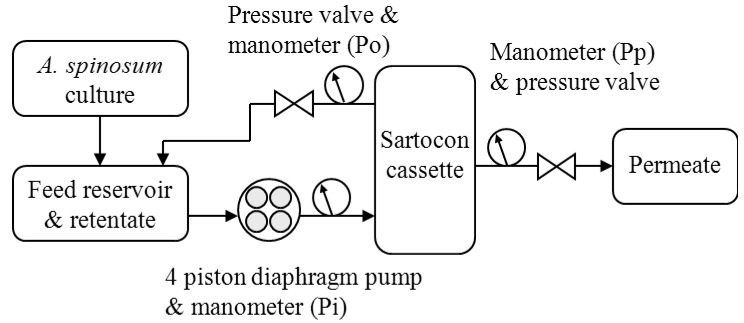
Schematic presentation of the tangential flow filtration for *A. spinosum* culture.

The trans-membrane pressure (TMP) for this system was defined by the following equation:

TMP = (Pi + Po)/2 − Pp

where Pi, Po and Pp are, respectively, the pressure at the inlet, outlet and permeate (Figure 3).

The pump was maintained at 70% of its capacity during filtration (8–11 L·min^−1^ depending on the pressure applied), and TMP was regularly adjusted to 0.3 bar as recommended by the manufacturer using pressure valves (Po and Pp). Thus, 200 L of *A. spinosum* culture was filtered to give less than 1 L of algal concentrate (retentate) and almost 200 L of permeate (Figure 3).

*Method 2*. Continuous centrifugation (Clara 20, Alfa Laval) was applied to separate the algae from the culture medium (70 L·h^−1^, 11,000 *g*, room temperature). 200 L of algal culture thus provided ~15 L of algal concentrate (retentate) and ~185 L of supernatant, from which AZAs were then extracted using methods 4 and 6 (see below).

### 3.5. Extraction of AZA1 and -2 from the Retentate and Permeate

Two procedures were tested for toxin extraction from the retentate:

*Method 3*. The algal concentrate was centrifuged (3500 *g*, 30 min, 4 °C) and collected as an algal paste. The analytical extraction procedure was scaled up [52], using three consecutive extractions of the algal paste with 5 mL of organic solvent per gram of algal paste. The combined extracts were evaporated using a rotary evaporator (Büchi Rotavapor R-200), weighed, and the residue was reconstituted in 5 mL methanol for analysis.

*Method 4*. The algal concentrate was sonicated (pulse mode, 20 min in ice, Bioblock Scientific, Vibra-cell 75115), 25 g of activated Diaion HP-20 polymeric resin was added, and gently agitated with the algal concentrate for 24 h (the optimum contact time as determined below) on a laboratory shaker (IKA Labortechnik, KS125 basic). The resin was recovered on 100 µm phytoplankton mesh, then washed with 1 L of Milli-Q water (Millipore, Integral 3 system), and placed in a glass column (3 × 60 cm) with a glass frit at the bottom. The toxin was eluted with three volumes of acetone (50 mL) at 1 mL·min^−1^. Subsequently, the extract was evaporated and reconstituted as above.

Two procedures were tested for extraction of AZAs from the permeate:

*Method 5*. Eight SPATT bags (solid phase adsorption toxin tracking) [53,55,56] each containing ~3 g of activated Diaion HP-20 resin were added to the permeate and gently agitated with a submerged pump over 72 h (72 h was the optimum, with 1, 15, 24 and 72 h having been tested). The SPATT bags were then recovered, opened, and the resin was washed and extracted as in method 4.

*Method 6*. A submerged pump (20 L·min^−1^) was placed in the permeate and used to circulate the permeate through a column containing 25 g of activated Diaion HP-20 resin for 72 h. The resin was then washed and extracted as above. This procedure was an adaptation of Rundberget *et al.* [37] developed for large scale extraction of microalgal biotoxins *in situ*.

Extraction procedures involving Diaion HP-20 polymeric resin were optimized in the present study to determine the best extraction yield. Four experiments were carried out. Acetone was chosen as eluent to reduce the risk of formation of methylated AZAs [52].

Procedures for the extraction of AZAs with HP-20 were optimized:

- The minimum amount of HP-20 resin required for efficient extraction was evaluated (in triplicate). Resin (1, 2.5, or 5 g) was placed in sonicated retentate (100 mL) for 24 h, recovered, packed in a glass column, and eluted with acetone (3 × 3 times the volume of the HP-20 resin), to give final volumes of 9, 22.5, and 45 mL, respectively.
- The effect of contact time (2, 6, 24 and 72 h) between the sonicated retentate (100 mL) and the HP-20 resin (2.5 g) was tested in triplicate.
- The volume of solvent required for elution was determined in triplicate experiments where 5 g batches of HP-20 resin were placed in sonicated retentate (100 mL) for 24 h, recovered, packed in a glass column, and eluted using different volumes of acetone ((a) 3 × 5 mL; (b) 3 × 10 mL; (c) 3 × 25 mL).
- Using the optimized procedures (2.5 g of resin, 24 h contact), adsorption efficiency was assessed by comparing the initial and final amounts of toxin in the sonicated retentate. The following elution procedures were then tested and the overall AZA recoveries determined (Table 3): (a) desorption using three successive soaks of acetone (3 × 7.5 mL) for 5 min; and (b) for 2 h [53]; (c) elution from a glass column (1 mL·min^−1^) using three successive additions of acetone (3 × 7.5 mL) [55].

### 3.6. LC-MS/MS Analysis at Ifremer, Nantes

The samples were analyzed by LC-MS/MS using an Agilent 1100 model coupled to a triple quadrupole mass spectrometer (API 2000, SCIEX, Applied Biosystems) for quantification of azaspiracids. Chromatography was performed with a Hypersil BDS C8 column (50 × 2 mm, 3 µm, Thermo scientific), with isocratic elution at 250 µL·min^−1^ for 10 min. The mobile phase was 100% water and acetonitrile–water (95:5), both containing 2 mM ammonium formate and 50 mM formic acid. The injection volume was 5 µL and the column and sample temperatures were 20 and 5 °C, respectively.

The declustering potential was 116 V, the entrance potential 10 V, the cell exit potentials 12 and 16 V, and the collision cell was 41 and 69 V for fragmentation 1 and 2, respectively. The electrospray ionisation interface (ESI) was operated using the following parameters: curtain gas: 30 psi; temperature: 450 °C; gas 1, 50 psi; gas 2, 50 psi; CAD gas, medium; ion spray voltage, 5500 V.

Azaspiracids were quantified against certified AZA1 standards obtained from the National Research Council Canada (NRCC). The two most intense product ions were selected with the following transitions: AZA1 *m/z* 842.5→824.5 and 842.5→672.4, and AZA2 *m/z* 856.5→838.5 and 856.5→672.4.

### 3.7. Analysis at the Marine Institute, Rinville and at NRCC, Halifax

#### 3.7.1. LC-MS/MS Analysis

Two LC-MS/MS systems were used in positive ion mode, both of which were equipped with a z-spray ESI source.

*Method A*. Recoveries were determined by quantitative analysis of fractions on a Waters 2695 LC coupled to a Micromass triple-stage quadrupole (TSQ) Ultima operated in multiple reaction monitoring (MRM) mode, with the following transitions: AZA1 *m/z* 842.5→824.5 and 842.5→672.4, AZA2 856.5→838.5 and 856.5→672.4. The cone voltage was 60 V and the collision voltage was 40 V, the cone and desolvation gas flows were set at 100 and 800 L/h, respectively, and the source temperature was 150 °C.

Binary gradient elution was used, with phase A consisting of water and phase B of 95% acetonitrile in water (both containing 2 mM ammonium formate and 50 mM formic acid) in a minor modification to the method of Quilliam *et al*. [60]. Chromatography was performed with a Hypersil BDS C8 column (50 × 2.1 mm, 3 µm, with a 10 × 2.1 mm guard column of the same stationary phase) (Thermo Scientific). The gradient was from 30% B, to 90% B over 8 min at 0.25 mL·min^−1^, held for 5 min, then held at 100% B at 0.4 mL·min^−1^ for 5 min, and returned to the initial conditions and held for 4 min to equilibrate the system. The injection volume was 5 µL and the column and sample temperatures were 25 °C and 6 °C, respectively.

*Method B*. Purity was initially assessed on a Micromass time-of-flight (QTof) Ultima coupled to a Waters 2795 LC by running MS scans (*m*/*z* 100–1000) using the same chromatographic conditions as method A. Identification of other AZA analogues was also determined by performing product ion scans, where the precursor ions were selected and then fragmented, for all the known AZA analogues (Table 1).

*Method C*. Qualitative analysis of fractions for AZAs was performed by flow injection analysis-MS/MS using a Micromass QTof Ultima coupled to a Waters 2795 LC. Samples (2 µL) were injected, using the 2795 autosampler, directly (no column) into the mass spectrometer monitoring for the precursor ions.

*Method D*. A concentrated sample (~500 µg·mL^−1^) was injected (1 µL) onto the semi preparative system (Shimadzu 10AVp) with photodiode array (PDA) detection (210 nm) using a Cosmosil C18 column (5 µm, 250 × 4.6 mm) eluted with acetonitrile–water (1:1, plus 2 mM ammonium acetate) at 1 mL·min^−1^. The column temperature was 30 °C.

*Method E*. An additional method employed to detect any strongly retained compounds (e.g., phthalates) used an analytical LC system (Shimadzu LC 10AVp) with PDA detection at 210 nm. The sample collected after the SPE step was injected (5 µL) onto a Vydac C18 column (10 µm, 250 × 4.6 mm, Grace) and eluted with methanol–water (9:1) at 1 mL·min^−1^, with the column temperature at 30 °C.

#### 3.7.2. NMR Spectroscopy

Purity was assessed by ^1^H NMR using a Bruker DRX-500 spectrometer. Chemical shifts were referenced to internal C*H*D_2_OH (3.31 ppm).

### 3.8. AZA1 and -2 Isolation from *A. spinosum* HP-20 Extract

HP-20 resin extracts from 1200 L of culture were combined, evaporated *in vacuo*, and partitioned between ethyl acetate (150 mL) and aqueous NaCl (1 M, 50 mL). The ethyl acetate fraction was evaporated to dryness *in vacuo* and the residue dissolved in ethyl acetate (20 mL), and ~2 g of silica gel was added. The sample was then carefully evaporated to dryness *in vacuo*, mixed to a fine powder and loaded onto a silica gel (6 g) column (6 × 4 cm). Vacuum-assisted elution was performed successively with hexane, ethyl acetate, ethyl acetate–methanol (9:1, 7:3, and 1:1), and methanol (30 mL of each, all containing 0.1% acetic acid except for hexane). The 7:3 ethyl acetate–methanol fraction, which FIA-MS/MS (Method C) showed to contain the AZAs, was evaporated *in vacuo*, and the sample in acetonitrile–water (6:4, plus 0.1% triethylamine) was loaded onto a column packed with Phenyl-Hexyl (19.9 × 2 cm). The sample was eluted with acetonitrile–water (7:13, plus 0.1% triethylamine) at 4 mL·min^−1^, and 5-mL fractions were collected. Appropriate fractions were combined (AZA1, fractions 15–18, and AZA2, fractions 19–25) based on FIA-MS/MS analysis.

Final purification of AZA1 and -2 was achieved by semi-preparative HPLC (Agilent 1200) with photodiode array (PDA) detection (210 nm) using a Luna C8 (5 µm, 250 × 10 mm, Phenomenex) column eluted with acetonitrile–water (1:1, plus 2 mM ammonium acetate) at 4 mL/min. The column temperature was 30 °C. Purified AZAs were recovered by evaporation to ~20% acetonitrile, loading on to SPE cartridges (Oasis HLB, 200 mg), washing with methanol–water (1:9, 10 mL) to remove the buffer, and eluting with methanol–water (4:6, 6:4, 8:2, 10:0, 20 mL each) with >95% of the AZAs eluting in the 8:2 fraction.

Purified samples were tested for phthalates (Method E) which, if present, were removed by partitioning the sample between methanol–water (4:1, 20 mL) and 20 mL of hexane. Removal of solvent by evaporation *in vacuo* afforded purified AZA1 (9.3 mg) and AZA2 (2.2 mg) as white solids.

The scheme of the isolation procedure of AZAs is presented in the ESM.

### 3.9. Reagents

Ifremer: acetone, acetonitrile (ACN) and dichlromethane (DCM) were obtained as HPLC grade solvents from JT Baker. Milli-Q water for the HPLC mobile phase was supplied by a Milli-Q integral 3 system (Millipore). Formic acid (Puriss quality), ammonium formate (Purity for MS) and Diaion HP-20 polymeric resin were from Sigma-Aldrich (Steinheim, Germany).

Marine Institute: all solvents (pestican grade) were purchased from Labscan (Dublin, Ireland). Sodium chloride (>99%), triethylamine (99%), ammonium acetate (>97%), ammonium formate (reagent grade), formic acid (>98%), and silica gel (10–40 µm, type H) were purchased from Sigma-Aldrich (Steinheim, Germany). Luna Phenyl-Hexyl (15 µm) was from Phenomenex (Cheshire, UK), and methanol-*d*_3_ (CD_3_OH, 99.5%) was from Cambridge Isotope Laboratories (MA, USA).

AZA1 and -2 certified reference materials (CRMs) were obtained from the NRC, Certified Reference Material Program (Halifax, NS, Canada).

### 3.10. Statistical Analysis

Data were expressed as mean ± standard deviation (SD). Statistical analyses were multi-factor ANOVA were differences were considered significant at *p* < 0.05. Statistical analyses were carried out using Statgraphics Centurion XV.I (StatPoint Technologies, Inc.). Before each ANOVA analysis, normality and equality of variance were tested. ANOVA was followed by multiple comparison (Fischer least significant differences) procedures to discriminate differences between values within each factor.

## 4. Conclusions

This study presents the first data on *A. spinosum* production in pilot scale photobioreactors. It demonstrates the ability of this small and fragile dinoflagellate to grow in this type of reactor. The effect of growth rate on AZA cell quota and toxin production outline the secondary metabolite character of AZAs. A dilution rate of 0.25 day^−1^ was found to yield the highest volume-specific toxin production per day. The system of two bioreactors coupled in series at higher dilution rate allowed for an AZA production rate of 475 µg·day^−1^ (19 µg·day^−1^·L^−1^). Under these conditions we obtained ~3 mg of AZAs in crude extracts over 12 days (8 days of culture, 1 day of filtration and 3 days of extractions). Further work is under way on the influence of environmental and nutritional parameters other than dilution rate on *A. spinosum* growth and toxin production.

The production of large volumes of *A. spinosum* necessitated the development of rapid procedures to concentrate and harvest algal cells for optimal toxin recover. Both continuous centrifugation and tangential flow filtration have been successfully applied in this study to concentrate *A. spinosum* culture. A simple and efficient procedure of extraction allowed for retrieval of toxins from both the retentate and the permeate to provide a crude *A. spinosum* extract rich in AZA and low in interfering matrix components.

Thanks to the high concentration of AZAs relative to the matrix in the extract, purification of AZA1 and -2 could be simplified to a 4-step procedure with recoveries of 75% and 70% for AZA1 and -2, respectively, a 1.5 fold increase when compared with isolation of AZAs from shellfish. In this study, 6 successive culture lots (1200 L harvested in total) enabled the recovery of 9.3 mg of purified AZA1 and 2.2 mg of purified AZA2.

Future production of AZAs using *A. spinosum* could use bioreactors at this scale, or at an even larger scale. Still, one limitation of the presently developed approach is the fact that only AZA1 and -2 may be produced using *A. spinosum*, yet a number of other analogues are also relevant to official control [9]. As a consequence of another recent study [61], which suggests some self-limitation of mussels exposed to live *A. spinosum*, our current work investigates the use of crude extracts of *A. spinosum* for the exposure of mussels with a view to producing shellfish metabolites of AZA1 and -2. Such studies are aimed at more efficient production of shellfish metabolites but may also contribute to the understanding of the uptake from the dissolved phase and metabolism of AZAs by mussels.

More generally, this work showed that even though *A. spinosum* and many other dinoflagellates are known to have slow growth rates and low maximum cell concentrations compared to many other micro-algae used in shellfish aquaculture, their culture in photobioreactors is a viable biotechnological approach to the production of toxins with applications in research and operational food safety surveillance programs. Furthermore, metabolite production in chemostat bioreactors is stable and predictable, and has the possibility to influence secondary metabolite production by using different culture conditions.

## Supplementary Files

Supplementary File 1: PDF-Document (PDF, 135 KB)
