# Supplementary material for: Production and Isolation of Azaspiracid-1 and -2 from Azadinium spinosum Culture in Pilot Scale Photobioreactors
Source: Mar Drugs. 2012 Jun 13;10(6):1360–82. doi: 10.3390/md10061360 (PMC3397445; doi:10.3390/md10061360)

Supplementary Materials

Figure S1. ¹H NMR spectrum of AZA1 following purification from *A. spinosum*.

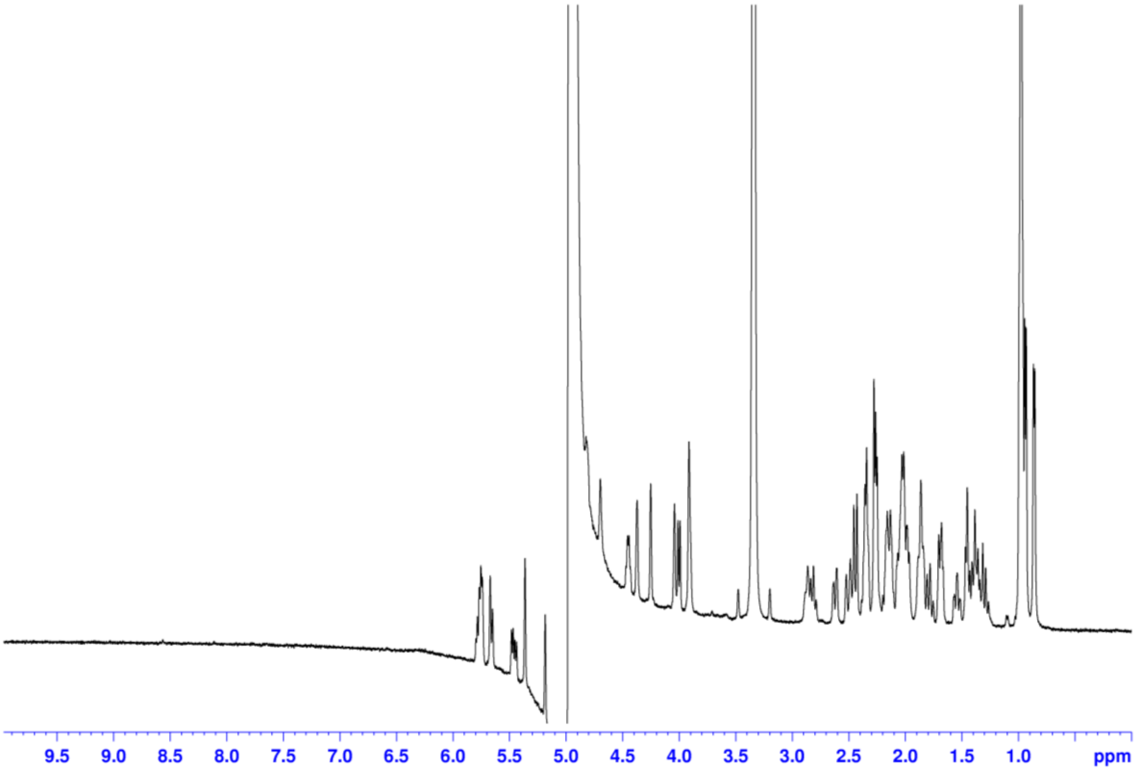

Figure S2. ¹H NMR spectrum of AZA2 following purification from *A. spinosum*.

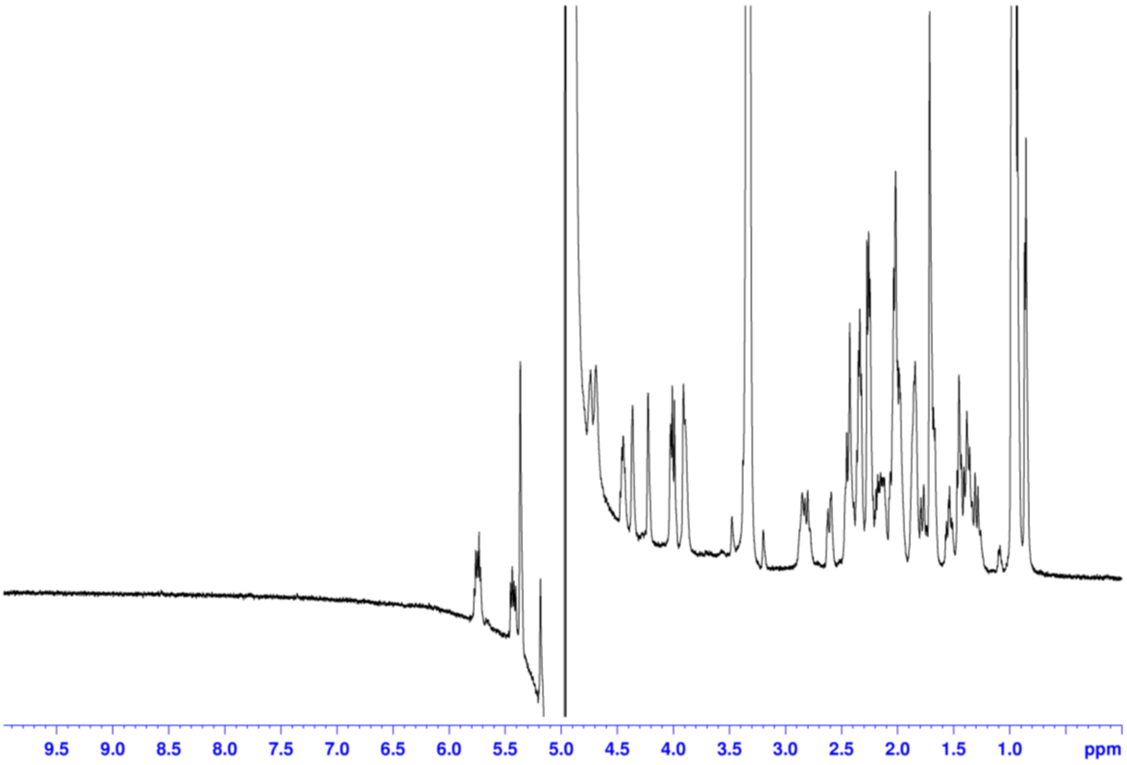

Figure S3. Procedure isolation of AZAs from *A. spinosum*.

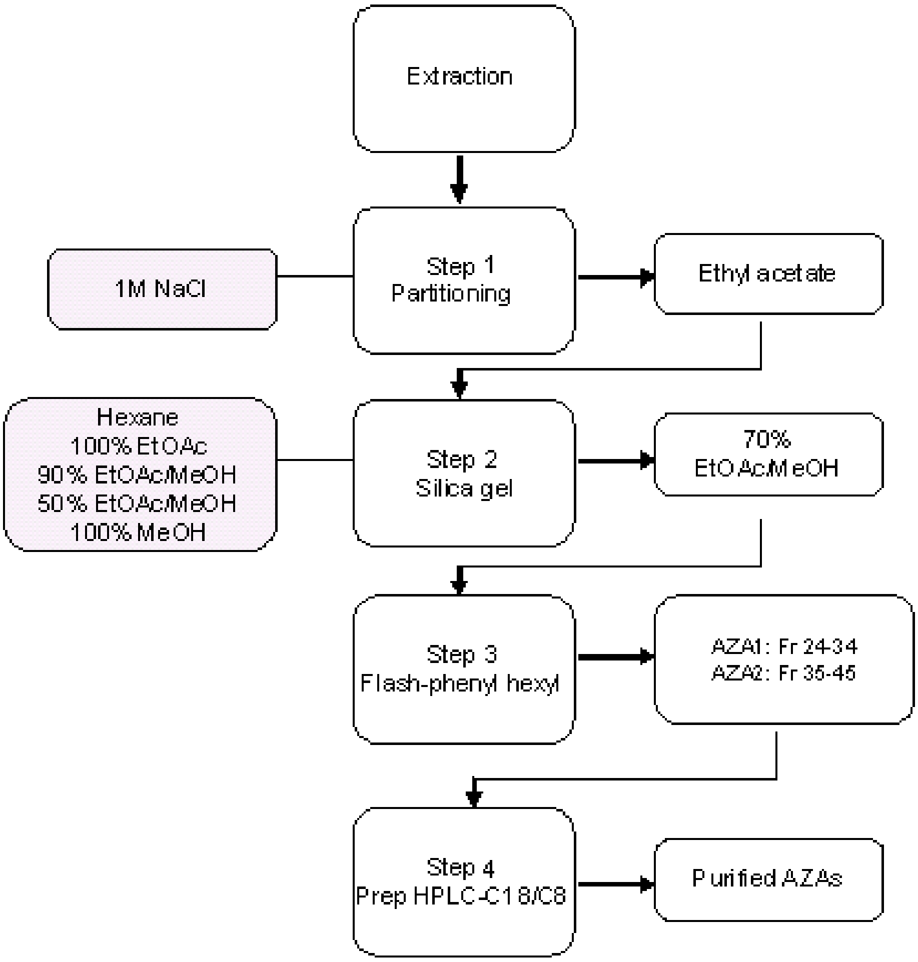

Supplement: Supplementary File 1: — PDF-Document (PDF, 135 KB) [file marinedrugs-10-01360-s001.pdf]
